# Cutaneous nerve distribution around first extensor compartment of the wrist: Clinical implications for ultrasound-guided injections^✰^

**DOI:** 10.1016/j.jpra.2024.01.013

**Published:** 2024-01-26

**Authors:** Hyun Il Lee, Hye-In Lee, Soonchul Lee, Wu-Chul Song

**Affiliations:** a Department of Orthopedic Surgery, Ilsan Paik Hospital, Inje University, 170, Juhwa-ro, Ilsanseo-gu, Goyang-si, Gyeonggi-do, 10380, Republic of Korea; b Department of Anatomy, Konkuk University School of Medicine, 120 Neungdong-ro, Gwangjin-gu, Seoul, 05029, Republic of Korea; c Department of Orthopedic Surgery, CHA Bundang Medical Center, CHA University School of Medicine, 11, Yatap-ro 65beon-gil, Bundang-gu, Seongnam-si, Gyeonggi-do, 13496, Republic of Korea; d Department of Oral Anatomy and Developmental Biology, College of Dentistry, Kyung Hee University, Seoul, 02447, Republic of Korea

**Keywords:** De Quervain's disease, First extensor compartment, Lateral antebrachial cutaneous nerve, Steroid injection, Superficial radial nerve

## Abstract

**Purpose:**

To evaluate the course of the cutaneous nerve regarding the first extensor compartment to determine whether the dorsal or volar approach is safer for local injection into the first extensor compartment guided by ultrasound.

**Methods:**

We dissected the radial side of the wrists from 28 cadavers (52 wrists). Four-points along the imaginary line were set: the styloid process and 1 cm, 2 cm, and 3 cm proximal to the styloid process. The numbers of superficial radial nerve (SRN) and lateral antebrachial cutaneous nerve (LACN) branches were counted, and distances from the imaginary line at these points and nerve diameters were recorded. Digital images were superimposed to observe overall distribution of cutaneous nerve.

**Results:**

There were means of 3.3 SRN and 0.9 LACN branches observed in each wrist. The mean number of both SRN and LACN branches was 2.3 on the dorsal side and 1.9 on the volar side. The superimposed images indicated that both the dorsal and volar sides comprised abundant cutaneous nerves and that their paths varied markedly between patients. However, we observed that larger nerves with meaningful diameters were more abundant on the dorsal than the volar side.

**Conclusion:**

There were similar numbers of cutaneous nerves on both the dorsal and volar sides; however, we observed greater abundance of thicker cutaneous nerves on the dorsal side, and these were closer to the reference line than on the volar side. This anatomical study suggests that the risk imposed to cutaneous nerves would therefore be reduced when injection on the volar side.

## Introduction

De Quervain's disease is a common stenosing tenosynovitis that affects tendons in the first extensor compartment as a degenerative condition.^1^ Severe pain and limitation of motion around the radial sides of the wrist and thumb restricts daily life activities, and the symptoms can sometimes persist for quite a long time. First-line conservative treatment consists of rest, activity modification, and oral analgesic or nonsteroidal anti-inflammatory agent administration. If sufficient conservative treatment fails, local corticosteroid injection is commonly provided before considering surgical treatment.

Local corticosteroid injection around the first extensor compartments is very effective in relieving symptoms.^2^^,^^3^ However, several adverse events or complications after an injection can sometimes occur, such as skin atrophy, direct puncture, or irritation to nearby cutaneous nerves.^4^ Skin atrophy or thinning would become a complaint in young females who are sensitive to their appearance.^5^, ^6^, ^7^ To reduce the risk of this adverse event, ultrasound-guided injection has become popular.^8^^,^^9^ Such an injection has the potential advantage of the corticosteroid more accurately targeting an anatomical site rather than just the subcutaneous area.^9^^,^^10^ Another advantage is the ability to identify the septum between extensor pollicis brevis tendon (EPB) and abductor pollicis longus tendon (APL), making it possible to perform injections specifically guided into the EPB or APL compartments.^11^^,^^12^

**Figure 1:**
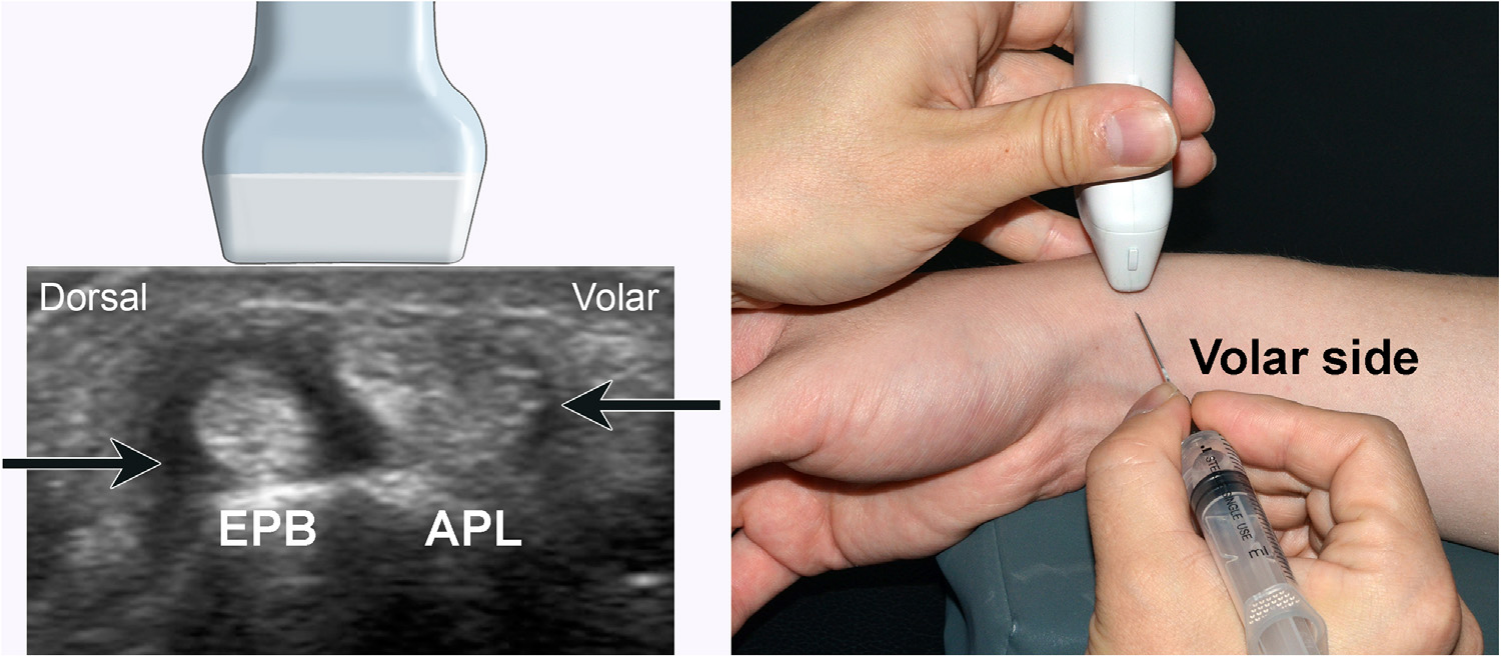
Ultrasound-guided steroid injection in De Quervain's disease. **A**. An ultrasound probe was located vertically along the path of the abductor pollicis longus muscle (APL) and extensor pollicis brevis muscle (EPB) to provide a short-axis view of the tendon. The needle for local steroid injection could be introduced from either the dorsal or volar side (arrow). **B**. This image shows the actual practice of observing the short-axis view using the probe, with the needle approaching from the volar side.

When we performed ultrasound-guided injections, long- or short-axis views were utilized based on the preference of the physician. In our practice, we prefer to inject EPB and APL compartment separately, a procedure that is performed when the ultrasound probe is positioned at right angles to the tendon; that is, on the short axis (Figure 1). In this situation, the needle could be inserted via a dorsal or volar approach into the first extensor compartment to provide an ‘in-plane’ view. The presence of more abundant nerves, either dorsal or volar, may be related to the risk of nerve damage. There has been extensive research on the distribution of the superficial branch of the radial nerve (superficial radial nerve [SRN]) around the radial side of the wrist, most of which focused on surgery such as first extensor compartment release or arthroscopy.^13^, ^14^, ^15^, ^16^ No previous study has localized cutaneous nerves around the first extensor compartment for injections. The purpose of this study was therefore to identify all cutaneous nerves around the first extensor compartment associated with injection in De Quervain's disease, so to find out which direction of injection is safer for avoiding cutaneous nerve injury.

## Materials and methods

Fifty-two wrists were dissected from 28 cadavers (15 females and 13 males, age range: 66∼98 years, mean age: 82.0 years). We dissected the wrists bilaterally in 24 cadavers and unilaterally in 4. Cases with a history of wrist surgery or nonneutral position due to postmortem rigor were excluded. This study was performed in accordance with the principles of the Declaration of Helsinki, and appropriate consents and ethical approval were obtained from the Ilsan Paik Hospital (ISPAIK 2023-11-016-001). The authors made every effort to follow all local ethical guidelines and laws that pertained to the use of cadaveric donors in anatomical research.^17^

**Figure 2:**
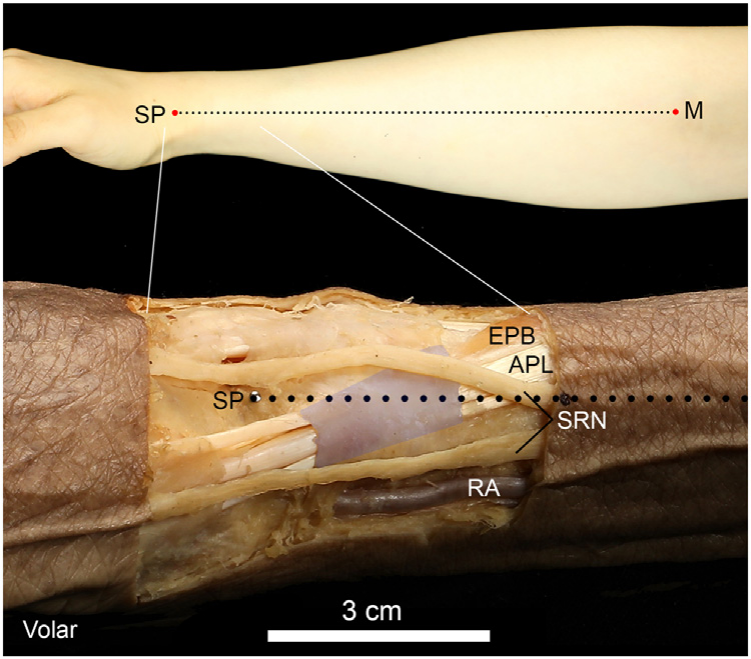
The reference line for the measurements is shown in the upper panel, and a photograph of a dissected wrist is shown in the lower panel. The first extensor retinaculum was painted with a darker color. The reference line is depicted as a dot. Two superficial radial nerve (SRN) branches were present on both the volar and dorsal sides of this specimen. SP, styloid process of the radius; M, midpoint of the antecubital fossa; RA, radial artery; APL, abductor pollicis longus muscle; EPB, extensor pollicis brevis muscle; SRN, superficial radial nerve.

An imaginary line between the middle of the antecubital fossa (midpoint between the lateral and medial epicondyles) and the tip of the styloid process of the radius (SP) was used as the reference line (Figure 2). This reference line was selected because this posture was the same position as that when the injection was performed. We made a rectangular incision border around the SP. The incision line ran vertically 2 cm toward the dorsal and volar directions from the reference line, and 1 cm and 3 cm horizontally toward the distal and proximal side far from the SP, respectively. The skin around the lateral wrist was removed, and minimal subcutaneous dissection near the SP was carefully performed to avoid injury to the SRN and lateral cutaneous nerve of the forearm (lateral antebrachial cutaneous nerve [LACN]). Finally, the EPB and APL tendons with surrounding first extensor compartments were identified (Figure 2). The LACN was often located more superficially than the SRN, and in cases where it was difficult to discriminate between the SRN and LACN, the nerve was traced to the proximal side to determine whether it was the SRN or LACN.

**Figure 3:**
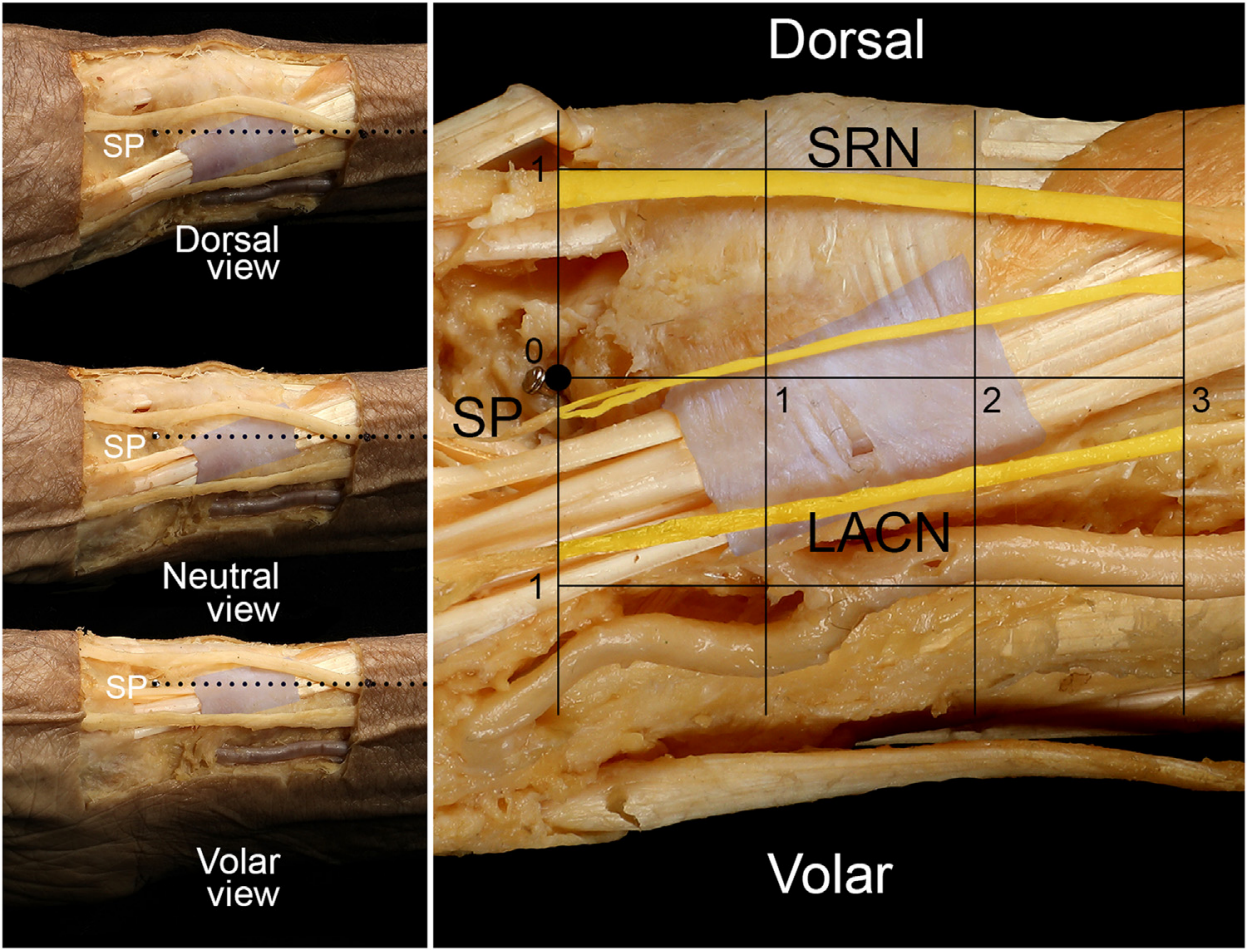
Photographs were taken from three different angles to minimize anatomical distortion caused by 3D differences in depth. After obtaining dorsal, neutral, and volar views as in the left panel, we counted the numbers of nerves, distances from the reference line, and diameters. Four points were set as SP, #1, #2, and #3 (right panel). The grid was included to help in measuring each parameter. SP, styloid process; LACN, lateral antebrachial cutaneous nerve; SRN, superficial radial nerve. Unit: cm.

**Figure 4:**
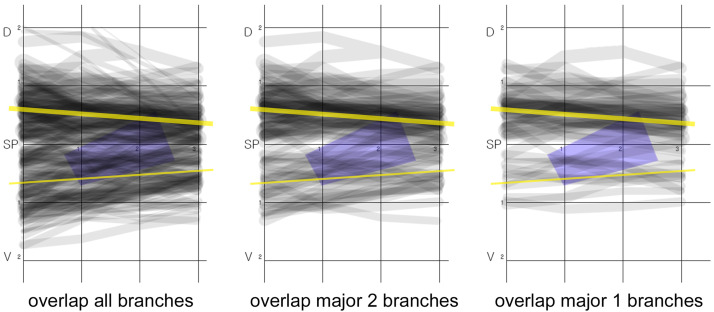
All of the cutaneous nerves were superimposed in a single image with a grid (left panel). The reference line is in the center of the grid passing through the SP, and a representative nerve with its mean diameter and distance from the reference line is depicted on both the dorsal (D) and volar (V) sides (yellow line). The first extensor compartment is indicated by a blue trapezoidal rectangle. In the central panel, only the main two branches of each cadaver were overlapped to simplify observations of the nerve distribution. In the right panel, only the main single branch was overlapped, and it is clear that the main thicker nerve is more abundant on the dorsal side. SP, styloid process. Unit: cm.

Because the measurement area was very small, we decided to perform indirect measurements on photographs rather than measuring the parameter directly on the cadaver. Each of the neutral, dorsal, and volar views were photographed as parallel as possible to the scale ruler (Figure 3). The grid was placed at 1-cm intervals in the proximal, dorsal, and volar directions using a computer program (Adobe Photoshop CS6, Adobe System, San Jose, CA, USA) (Figure 3). Four points set along the reference line were the SP and 1 cm, 2 cm, and 3 cm proximal to the SP, and were denoted as points SP, #1, #2, and #3, respectively. The numbers of SRN and LACN branches were counted, and distances from the imaginary line were measured at points SP, #1, #2, and #3. Dorsal and volar distances were measured from the reference line at each set point. Cutaneous nerve diameters were also recorded. If the cutaneous nerve was on the dorsal or volar side, the distance from the reference line was expressed as a positive or a negative number, respectively. If the cutaneous nerve crossed the reference line and ran from the dorsal to volar side or vice versa, the values ​​of the four points (distances from the reference line) were summed, and if the value was positive or negative, it was considered to be located on the dorsal or volar side, respectively. Left-side samples were flipped to the right side so that they could be overlaid. All SRNs and LACNs were redrawn in the grid to consider their diameters and locations, and they were superimposed to give a complete view of the entire nerve in a single image (Figure 4). The superimposition of more nerves resulted in a deeper color.

Comparisons of the number, distance, and diameter between the nerves located in the dorsal and volar sides were performed using Student's *t*-test.

## Results

There was a mean of 4.2 cutaneous nerve branches in the investigated area (SD 1.7, range 1–8). Among them, the mean number of SRNs was 3.3 (SD 1.2, range 1–6) and that of LACNs was 0.9 (SD 1.0, range 0–3). The number of dorsal-located nerves was 2.3 (SD 1.2, range 0–6) and that of volar-located nerves was 1.9 (SD 1.1, range 0–4). There was no significant difference in the distribution of nerves between the volar and dorsal sides (*p* = 0.105). The distributions of SRNs and LACNs in the dorsal or volar sides are listed in Table 1.

**Table 1:** Mean numbers of cutaneous nerves on the dorsal and volar sides.

	SRN	LACN	Total (SRN+LACN)
Dorsal	1.8	0.5	2.3
Volar	1.5	0.5	1.9
Total (dorsal + volar)	3.3	0.9	4.2

SRN, superficial radial nerve; LACN, lateral antebrachial cutaneous nerve.

Mean distance from reference line was 5.5 mm, 4.9 mm, 4.4 mm, and 3.9 mm from the SP, 1 cm, 2 cm, and 3 cm on dorsal side, respectively (larger yellow lines in Figure 4). Mean distance from reference line was 6.6 mm, 5.9 mm, 5.3 mm, and 4.4 mm from SP, 1 cm, 2 cm, and 3 cm on volar side, respectively (smaller yellow lines in Figure 4). The distances differed significantly only at point #1 (*p* = 0.04, Table 2). We also observed the trend that the volar side nerve was slightly further from the dorsal side at points SP and #2, but these differences were not statistically significant (*p*>0.1).

**Table 2:** Distances between cutaneous nerves and the imaginary reference line.

	SP	#1	#2	#3	Total^†^
Dorsal	5.5 ± 4.0	4.9 ± 3.7	4.4 ± 3.4	3.9 ± 2.9	4.7 ± 3.6
Volar	6.6 ± 4.3	5.9 ± 3.6	5.3 ± 3.4	4.4 ± 3.2	5.6 ± 3.7
*p*	0.079	0.046	0.067	0.225	<0.001

Mean±SD, unit: mm.

SP, styloid process of the radius.

The diameters at each of the four points are listed in Table 3. The mean cutaneous nerve diameter was 1.7 mm on the dorsal side and 1.4 mm on the volar side (*p*<0.001). The cutaneous nerve at each point was thicker on the dorsal side than on the volar side except for at point SP (Table 3). The mean diameter of the SRN was larger than that of the LACN (1.8 mm versus 0.8 mm, *p*<0.001).

**Table 3:** Diameters of cutaneous nerves on the dorsal and volar sides.

	SP	#1	#2	#3	Total†
Dorsal	1.4 ± 0.7	1.8 ± 0.9	1.8 ± 0.9	1.8 ± 0.9	1.7 ± 0.9
Volar	1.3 ± 0.7	1.3 ± 0.7	1.5 ± 0.7	1.5 ± 0.5	1.4 ± 0.7
*p* value	0.505	<0.001	0.007	0.004	<0.001

Mean±SD, unit: mm.

† When all data are combined including points SP, #1, #2, and #3.

We superimposed all the nerves into a single image (Figure 4). When we selected just one or two major nerves (larger nerves) to simplify the visualization, it was visually evident that the cutaneous nerves were more abundant on the dorsal side than on the volar side (middle and right panels of Figure 4).

## Discussion

This study focused on visualizing all the cutaneous nerves around the first extensor compartment, paying particular attention to whether the nerves were located on the dorsal or volar side. The aim was to determine which side is safer to inject into. Fine anatomical dissections revealed a greater abundance of larger cutaneous nerves on the dorsal side. We demonstrated significant differences in the distances and diameters of nerves located on the dorsal side compared with the volar side. Superimposed images also indicated that cutaneous nerves were more abundant on the dorsal side than on the volar side. Given that the nerves on the dorsal side were located closer to the reference line in our anatomical study, it can be concluded that it could be safer to inject from the volar side.

However, the present findings do not necessarily demonstrate that injection through the dorsal side will be more dangerous in a real clinical situation. There are still no clinical data showing that the volar approach is safer than the dorsal approach. A clinical confirmatory study such as a prospective observatory study or a prospective randomized study is required to confirm the hypothesis generated from this anatomical study. One problem in performing such a clinical study is that the adverse event of puncturing a nerve is very rare, and many patients would therefore need to be recruited to confirm our hypothesis.

Indeed, in our practice we have experienced only one adverse event of puncturing or irritating the cutaneous nerve when we injected the needle through the dorsal side. That patient suffered from severe nerve-origin pain for more than 6 months, but the pain subsided after conservative treatment and skillful neglect. We planned this anatomical study after that event. We switched to injections on the volar side based on the current anatomical study. Since then, we have not experienced a nerve-puncturing event.

In 1992, Abrams et al. reported the distribution of SRN in the forearm and its surgical implications.^13^ They found that the branch nearest to the center of the first extensor compartment was located at a mean distance of 4 mm, which was similar to our average values. Their mean diameter of the SRN was 2 mm at the extensor retinaculum level, which was comparable to ours (1.8 mm). In 2004, Ikiz et al. also reported that the nearest branch of SRN was a mean of 5.4 mm from the first dorsal compartment.^15^ However, these two studies did not identify whether the location of the SRN was dorsal or volar relative to the first extensor compartment.

The two aforementioned reports investigated the full course of the SRN in the forearms of cadavers; however, Gurses et al. focused on the confined area of first extensor compartment in 2014.^18^ They investigated the two branches of the SRN (main branch and lateral dorsal digital branch [LDDB1]) and found that the main branch of the SRN was often located on the dorsal side of the first extensor compartment and it was farther from midpoint in distal area compared to proximal area (proximal, middle, and distal midpoints were 5 mm, 7 mm, and 9 mm from the midline, respectively). In contrast, the LDDB1 was on the volar side of the first extensor compartment and remained 2 mm from the midline along the path of first extensor compartment. These results were not consistent with ours that the dorsal cutaneous nerve was closer to the extensor compartment compared with the volar cutaneous nerve. This discrepancy could be due to Gurses et al. studying only two branches of the SRN. Other possible reasons are racial differences or differences in the reference lines. In 2018, Poublon et al. performed very similar study with ours regarding SRN and LACN distribution in surgical release of 1st extensor compartment release.^19^ They also tried to superimpose all the nerve data of 20 cadavers to visualize “average” arm using computer technique. They demonstrated relatively safe zone as just above 1st extensor retinaculum and this gradient map would be helpful in performing surgery or injection. However, they do not measure the diameter and distance from midline of each nerve.

There have also been very few anatomical studies of the LACN at the wrist, because the LACN is arborized into the small terminal branches in the wrist. Most previous studies of the LACN were confined to the middle or proximal forearm area.^20^^,^^21^ A rare study of the distribution of the LACN in the wrist found that the LACN and SRN were distributed in the overlapping skin area in 75 % of cases.^22^ The LACN often ran parallel to the cephalic vein, and these two structures are commonly located in the dorsal side in close proximity to the anatomical snuffbox.^20^^,^^23^

There is controversy about the additional benefit of ultrasound-guided injections compared with anatomical landmark-based injections (namely blind injections). Ultrasound-guided injections are expected to be more effective, but there have been conflicting results.^2^^,^^8^, ^9^, ^10^^,^^12^^,^^24^^,^^25^ An additional benefit of ultrasonography was that we could determine whether the septum of the EPB was separated. A cadaveric study indicated that the accuracy of landmark-based injection was especially poor in cases with a separated septum in the EPB.^25^ Injections can be accurately targeted to both compartments or the EPB subcompartment when using ultrasonography.^24^

There were several limitations to our study. First, the 3D structure was not considered. The injection depth is important, but it was difficult to investigate. However, since the first extensor compartment is so superficial, it is considered that the effect of depth would be less significant. Second, the proximities of the radial artery and cephalic vein were not investigated. The probability of damage to blood vessels may differ slightly depending on whether an injection is performed on the volar or dorsal side. However, the radial artery was somewhat further away, so it was considered safe to enter from the volar side. Third, we used a predefined reference line instead of the first extensor compartment or retinaculum. This method was simpler in conducting the study, and the first extensor compartment was not far from our reference line (depicted in Figure 4). This reference line would also be easy to identify during clinical injections.

In conclusion, the cutaneous nerves around the first extensor compartment were more abundantly distributed on the dorsal side. According to our anatomical study, volar-side injection could be safer regarding the risk of nerve injury. However, the choice of injection side could be based on the preference of the physician until it is found that more clinical data confirm our hypothesis.

## Ethical approval statements

This study was performed in accordance with the principles of the Declaration of Helsinki, and appropriate consents and approval were obtained prior to the study commencing. The authors made every effort to follow all local ethical guidelines and laws that pertained to the use of cadaveric donors in anatomical research.

## Declaration of competing interest

The authors declare no competing interests.

## References

[1] Larsen C.G., Fitzgerald M.J., Nellans K.W., Lane L.B. (2021). Management of de Quervain tenosynovitis: A critical analysis review. JBJS Rev.

[2] Hajder E., de Jonge M.C., van der Horst C.M., Obdeijn M.C. (2013). The role of ultrasound-guided triamcinolone injection in the treatment of de Quervain's disease: Treatment and a diagnostic tool?. Chir Main.

[3] Peters-Veluthamaningal C., van der Windt D.A., Winters J.C., Meyboom-de Jong B. (2009). Corticosteroid injection for de Quervain's tenosynovitis. Cochrane Database Syst Rev.

[4] Ilyas A.M., Ast M., Schaffer A.A., Thoder J. (2007). De Quervain tenosynovitis of the wrist. J Am Acad Orthop Surg.

[5] Brinks A., Koes B.W., Volkers A.C., Verhaar J.A., Bierma-Zeinstra S.M. (2010). Adverse effects of extra-articular corticosteroid injections: A systematic review. BMC Musculoskelet Disord.

[6] Liang J., McElroy K. (2013). Hypopigmentation after triamcinolone injection for de Quervain tenosynovitis. Am J Phys Med Rehabil.

[7] Venkatesan P., Fangman W.L. (2009). Linear hypopigmentation and cutaneous atrophy following intra-articular steroid injections for de Quervain's tendonitis. J Drugs Dermatol.

[8] Kang J.W., Park J.W., Lee S.H. (2017). Ultrasound-guided injection for De Quervain's disease: Accuracy and its influenceable anatomical variances in first extensor compartment of fresh cadaver wrists. J Orthop Sci.

[9] Bing J.H., Choi S.J., Jung S.M. (2018). Ultrasound-guided steroid injection for the treatment of de Quervain's disease: An anatomy-based approach. Skeletal Radiol.

[10] Kume K., Amano K., Yamada S. (2012). In de Quervain's with a separate EPB compartment, ultrasound-guided steroid injection is more effective than a clinical injection technique: A prospective open-label study. J Hand Surg Eur Vol.

[11] Yeom J.W., Koh K.H., Park M.J. (2021). Modified staged Finkelstein test for the identification of intracompartmental septum in patients with De Quervain's disease. J Hand Surg Asian Pac Vol.

[12] Jung H.S., Baek S.H., Lee J.S. (2022). Is a steroid injection in both compartments more effective than an injection in the extensor Pollicis Brevis subcompartment alone in patients with de Quervain Disease? A randomized, controlled trial. Clin Orthop Relat Res.

[13] Abrams R.A., Brown R.A., Botte M.J. (1992). The superficial branch of the radial nerve: An anatomic study with surgical implications. J Hand Surg Am.

[14] Huanmanop T., Agthong S., Luengchawapong K. (2007). Anatomic characteristics and surgical implications of the superficial radial nerve. J Med Assoc Thai.

[15] Ikiz Z.A., Ucerler H. (2004). Anatomic characteristics and clinical importance of the superficial branch of the radial nerve. Surg Radiol Anat.

[16] Kilic A., Kale A., Usta A. (2009). Anatomic course of the superficial branch of the radial nerve in the wrist and its location in relation to wrist arthroscopy portals: A cadaveric study. Arthroscopy.

[17] Iwanaga J., Singh V., Takeda S. (2022). Standardized statement for the ethical use of human cadaveric tissues in anatomy research papers: Recommendations from Anatomical Journal Editors-in-Chief. Clin Anat.

[18] Gurses I.A., Coskun O., Gayretli O., Kale A., Ozturk A. (2014). The relationship of the superficial radial nerve and its branch to the thumb to the first extensor compartment. J Hand Surg Am.

[19] Poublon A.R., Kleinrensink G.J., Kerver A., Coert J.H., Walbeehm E.T. (2018). Optimal surgical approach for the treatment of Quervains disease: A surgical-anatomical study. World J Orthop.

[20] Beldner S., Zlotolow D.A., Melone C.P., Agnes A.M., Jones M.H (2005). Anatomy of the lateral antebrachial cutaneous and superficial radial nerves in the forearm: A cadaveric and clinical study. J Hand Surg Am.

[21] Hassan D.M., Johnston G.H. (1999). Safety of the limited open technique of bone-transfixing threaded-pin placement for external fixation of distal radial fractures: A cadaver study. Can J Surg.

[22] Mackinnon S.E., Dellon A.L. (1985). The overlap pattern of the lateral antebrachial cutaneous nerve and the superficial branch of the radial nerve. J Hand Surg Am.

[23] Steinberg B.D., Plancher K.D., Idler R.S. (1995). Percutaneous Kirschner wire fixation through the snuff box: An anatomic study. J Hand Surg Am.

[24] Leversedge F.J., Cotterell I.H., Nickel B.T. (2016). Ultrasonography-guided de Quervain injection: Accuracy and anatomic considerations in a cadaver model. J Am Acad Orthop Surg.

[25] Mirzanli C., Ozturk K., Esenyel C.Z. (2012). Accuracy of intrasheath injection techniques for de Quervain's disease: A cadaveric study. J Hand Surg Eur Vol.

